# A Person-Centred Approach to Organ Donation Decision-Making After Brain Death: A Case Report

**DOI:** 10.7759/cureus.100918

**Published:** 2026-01-06

**Authors:** Maria Kitsiou, Polyxeni Tsiokanou

**Affiliations:** 1 Department of Nursing, University of Ioannina, Ioannina, GRC

**Keywords:** brain death, end-of-life care, family decision-making, intensive care unit, organ donation, person-centred nursing

## Abstract

Organ donation following brain death represents a complex and emotionally charged process that extends beyond biomedical decision-making and is embedded within the broader context of end-of-life care in the intensive care unit. Although brain death is legally and medically recognised as death, families often experience significant emotional, cognitive, and existential challenges when asked to consider organ donation. We present the case of a 57-year-old man who progressed to death by neurologic criteria following severe hypoxic-ischemic injury. The case focuses on the family approach and the decision-making process surrounding organ donation. Initial difficulties in understanding the diagnosis, religious concerns, emotional distress, and the need for time and presence at the bedside were central to the family’s experience. Through repeated, empathetic communication, respect for the family’s spiritual beliefs, and the facilitation of meaningful farewell rituals, the healthcare team supported the family in reaching a consensual, values-aligned decision. The discussion analyses the case using the Person-centred Nursing Framework, highlighting how person-centred prerequisites, care environment, and processes contributed to trust, shared decision-making, and meaning-making.

## Introduction

Death by neurologic criteria, commonly referred to as brain death, is a legally and medically accepted definition of death in many countries and is characterised by the irreversible cessation of all functions of the brain, including the brainstem [1]. Advances in life-sustaining technologies have necessitated a neurologic definition of death, as cardiorespiratory function may be artificially maintained despite permanent loss of consciousness and the capacity for spontaneous breathing [1]. Contemporary philosophical and clinical analyses support the view that brain death represents the death of the human organism, even though certain biological functions may persist with mechanical support, emphasising the irreversible loss of consciousness and spontaneous respiration as defining elements [2].

Organ donation following brain death constitutes a complex process that extends beyond strictly medical boundaries and is embedded within the broader context of end-of-life care in the intensive care unit (ICU). Although brain death is internationally recognised as the definitive and irreversible death of the individual, families' understanding and acceptance of this diagnosis are often accompanied by intense emotional, cognitive, and existential challenges, particularly when they are asked to make decisions regarding organ donation [3,4].

The approach to families regarding organ donation takes place at an exceptionally vulnerable moment, characterised by acute grief, the need to find meaning in loss, and the effort to comprehend complex and often unfamiliar medical information. Contemporary ethical and clinical literature emphasises that this process should not be perceived as an act of "organ acquisition," but rather as an integral component of high-quality end-of-life care, grounded in respect, compassion, and the safeguarding of family autonomy [3,5].

The literature consistently highlights that the manner in which discussions about organ donation are conducted, including the timing, language, and attitudes of healthcare professionals, significantly influences both families' decisions and their subsequent experience and satisfaction with those decisions. Families who perceive pressure, coercion, or a process driven primarily by the demand for organs are more likely to experience ambivalence, emotional distress, or future dissatisfaction, regardless of their final decision [3,5].

Furthermore, a greater understanding of the concept of brain death, being given adequate time for decision-making, and receiving emotional support from healthcare professionals and the family's social environment are associated with greater acceptance of the decision and a lower likelihood of subsequent regret. The stability of family decisions regarding organ donation appears to be strengthened when the decision-making process occurs within a climate of trust, without pressure, and when families feel that their values, beliefs, and needs are acknowledged and respected [4].

In addition, the opportunity for presence, farewell, and meaningful physical contact with the patient before donation has been described as a key element in families' ability to attribute positive meaning to the experience and to integrate the donation decision into their grieving process [6]. The attitudes, education, and communication skills of ICU healthcare professionals are therefore recognised as critical factors influencing the quality of the organ donation process following brain death [5,6].

Within this context, the present case report describes an organ donation process following brain death, focusing on the family approach and the decision-making process. The analysis aims to illustrate how a person-centred approach can support both families and healthcare professionals, contributing to a care experience that preserves dignity, respect, and meaning, even in the most challenging moments.

## Case presentation

A 57-year-old man with a known history of amyotrophic lateral sclerosis (ALS) had progressively developed dysarthria and dysphagia. During feeding at home, he experienced an aspiration episode followed by acute respiratory distress. Emergency medical services were contacted; however, before their arrival, the patient suffered a cardiac arrest. He was transferred to the emergency department, where return of spontaneous circulation was achieved after approximately 35 minutes of prolonged cardiopulmonary resuscitation.

Prolonged hypoxia resulted in severe hypoxic-ischemic encephalopathy. The patient was admitted to the ICU, where his neurological condition remained extremely poor. Neuroimaging findings were consistent with irreversible brain injury. A clinical neurological examination, performed in the absence of sedative medications, revealed no responsiveness and a complete lack of all brainstem reflexes.

Following an initial discussion with the family regarding the severity of the patient's condition and the irreversible nature of the neurological injury, the medical and nursing team proceeded with the required clinical assessments for the determination of death by neurologic criteria. After completion of the protocol, the patient was diagnosed with death by neurologic criteria (brain death), in accordance with established medical and legal standards.

The organ donor coordinator (ODC) (who was by profession a nurse) was subsequently notified, and a family meeting was arranged. The patient's family consisted of his wife and four children. Only one son regularly attended visiting hours and medical updates. The ICU director, the attending physician, the named nurse, and the ODC participated in the first meeting.

During the initial disclosure, both the patient's wife and son demonstrated difficulty understanding the diagnosis, as they associated death exclusively with cessation of cardiac activity. An extended discussion followed, during which the concept of brain death was explained in detail. Gradually, a greater level of understanding appeared to develop. Within the same initial meeting, and after extensive explanation of the diagnosis, the option of organ donation was introduced. The patient's wife requested time to consider the decision and expressed concern regarding the position of the Greek Orthodox Church, as the family identified as deeply religious. The ODC provided additional information beyond the usual organ donation process, which included the Greek Orthodox Church's position on organ donation, and encouraged her to consult her spiritual advisor. A follow-up meeting was scheduled for 24 hours later.

At the second meeting, the medical team reiterated the explanation of death by neurologic criteria. The discussion fluctuated, with repeated questions regarding the persistence of cardiac activity and the possibility of a miracle. The team clarified once more that the patient was legally and medically deceased and explained that, according to institutional and legal practice, withdrawal of life-sustaining treatment would follow unless organ donation was pursued or a short extension was required to allow family members to say goodbye.

As the family was still in the process of emotionally processing both the loss and the impending decision, the wife and son were offered the opportunity to spend additional time at the patient's bedside in the ICU, which they accepted. They remained with the patient for approximately one hour, during which they sat beside the bed, held his hand, and cried, expressing their farewell and engaging with the emotional reality of the loss. Following this time at the bedside, they requested another meeting with the team and informed them of their decision to proceed with organ donation.

After consent for organ donation was provided, the healthcare team offered the patient's wife the opportunity for a priest to be present to say a prayer, acknowledging the importance of the family's spiritual and religious needs. This offer was received with relief and appreciation, as it represented a meaningful final wish for the family during the farewell process. The patient's wife reported that she had spoken with her spiritual advisor, who confirmed that the Church supports organ donation when the diagnosis is clear. She also noted that discussions within her social environment influenced her decision-making. The family confirmed that all children agreed with the decision. After signing the consent form, the wife shared a profoundly impactful family experience, explaining that a close family member had previously died from renal failure while awaiting transplantation. This experience reinforced her belief that organ donation could provide meaning and moral value to her husband's death. The patient’s clinical course and key decision-making points are summarised in Table 1.

**Table 1:** Sequence and key elements of family meetings during the organ donation decision-making process The table summarizes the timing, participants, family needs, and person-centred interventions that supported shared decision-making and meaning-making.

Time since ICU admission	Participants	Family’s expressed concerns / needs	Team interventions and responses
Initial meeting (Day 1)	ICU director, attending physician, named nurse, organ donor coordinator, wife, son	Difficulty understanding brain death; association of death with cardiac arrest	Repeated, detailed explanation of brain death; empathetic communication; time allowed for questions
Follow-up meeting (Day 2)	Same team members	Religious concerns; uncertainty regarding the Church’s position; emotional distress	Provision of information on the Greek Orthodox Church’s stance; encouragement to consult a spiritual advisor
Bedside presence	Wife, son, nursing staff	Need for time, presence, and farewell	Facilitated bedside presence; physical closeness; ongoing emotional support
Final meeting	ICU team, organ donor coordinator, family	Decision-making; search for meaning	Supportive discussion; respect for family values; confirmation of a consensual decision

## Discussion

This case highlights how organ donation following brain death can be experienced as a profoundly human, relational, and meaning-making process when approached through a person-centred lens. Rather than focusing solely on procedural or biomedical aspects, the care provided to the family reflected core principles of the Person-centred Nursing Framework (PCNF), particularly those related to prerequisites, the care environment, and person-centred processes. The PCNF is a theoretical nursing framework and not a measurement tool, scale, or scoring system, and was applied analytically to interpret the case findings [7]. Within the PCNF, prerequisites refer to the values, beliefs, and professional competence that nurses bring to the caring encounter. In this case, the healthcare team's approach demonstrated respect for personhood, emotional sensitivity, and a moral commitment to the family's experience. The willingness to remain present, listen attentively, and respond to the family's emotional cues reflects the PCNF emphasis on knowing the person and engaging authentically [7].

The family's visible distress, expressed through prolonged bedside presence, physical touch, and shared grieving behaviours, required nurses to move beyond task-oriented communication. The ability of the staff to tolerate silence, allow emotional expression, and avoid rushing the decision-making process aligns with previous literature highlighting the importance of emotional containment and relational competence during end-of-life and donation discussions [8]. The PCNF recognises that person-centred practice is enabled or constrained by the care environment. In this case, the ICU environment was intentionally shaped to support trust, psychological safety, and dignity. Allowing family members to remain with the patient, sit beside him, hold his hand, and openly express grief created a care environment that acknowledged relational needs alongside clinical realities.

Such practices resonate with evidence suggesting that families' experiences of organ donation are significantly influenced by whether they feel respected, unpressured, and emotionally supported throughout the process [9,10]. The absence of coercion and the provision of time for reflection contributed to a sense of control and ownership over the decision, reinforcing the stability of consent.

Person-centred processes within the PCNF include working with beliefs and values, shared decision-making, and providing holistic care. A defining moment in this case was the team's response to the family's religious needs. The proactive offer of information on the Church's position on organ donation, the space to discuss organ donation with the spiritual advisor, and the invitation of a priest, following consent for donation, are all actions that acknowledge spirituality as a core component of personhood rather than an adjunct to care. These actions supported the family's meaning-making process and transformed the donation experience into one that aligned with their values and final wishes. Previous studies have shown that recognising spiritual and cultural dimensions can help families integrate organ donation into their grieving process and reduce long-term emotional burden [7,9].

Importantly, the opportunity for farewell and physical closeness before organ procurement functioned as a therapeutic intervention in itself. Within a person-centred framework, such moments are not optional extras but essential caring practices that uphold dignity and relational continuity at the end of life. Although outcomes in the PCNF are often discussed in terms of patient and staff experience, this case illustrates how families also experience outcomes that extend beyond the immediate clinical encounter. The family's reported sense of gratitude and emotional relief suggests that the person-centred approach contributed to a more integrated and less traumatic donation experience.

Consistent with previous research, when families perceive that care is grounded in compassion, transparency, and respect, they are more likely to experience peace with their decision, regardless of the emotional difficulty involved [8,9]. 

The application of the PCNF to the present case is summarized in Table 2.

**Table 2:** Interpretive application of the Person-centred Nursing Framework (PCNF) to the case findings This table presents the authors' interpretive application of the PCNF, based on the analysis of the present case. The framework is used analytically to illustrate how person-centred prerequisites, care environment, processes, and outcomes were reflected in the family approach and decision-making process. Reference: McCance and McCormack, 2025 [7]

PCNF Domains	Key Concepts	Application in the Present Case
Prerequisites	Professional competence, values, and knowing the person	The healthcare team demonstrated emotional sensitivity, respect for personhood, and moral commitment by allowing time, listening to concerns, and responding to the family's emotional and spiritual needs.
Care Environment	Trust, psychological safety, and supportive context	The ICU environment was modified to allow prolonged bedside presence, physical closeness, and privacy, fostering trust and dignity during end-of-life decision-making.
Person-centred Processes	Shared decision-making, working with beliefs and values, engagement	Repeated meetings, avoidance of pressure, acknowledgement of religious beliefs, and facilitation of spiritual rituals supported shared, values-aligned decision-making.
Outcomes	Meaning-making, satisfaction, and acceptance	The family reported emotional relief and attributed positive meaning to organ donation, integrating the decision into their grieving process.

Clinical implications

This case underscores the importance of embedding the PCNF into organ donation practices within the ICU. Nurses and ODCs should be supported in developing relational, emotional, and ethical competencies alongside technical skills. Structured opportunities for presence, farewell, and spiritual support should be recognised as integral components of high-quality end-of-life and donation care. Furthermore, organisational cultures must enable flexibility, reflective practice, and emotional labour, ensuring that person-centred values are sustained even within high-pressure critical care environments [7,10].

As an interpretive case report, the analysis reflects the authors’ clinical and theoretical interpretation of the events and may not be generalizable.

## Conclusions

This report illustrates how organ donation after brain death can be experienced not merely as a medical or procedural process, but as a deeply relational and meaning-oriented event when approached through the lens of the PCNF. The family's journey toward consent was shaped by repeated, compassionate communication, respect for emotional vulnerability, acknowledgement of spiritual needs, and the provision of time and space for farewell and presence.

Applying person-centred principles allowed healthcare professionals to support the family's meaning-making process, transforming a potentially traumatic experience into one aligned with their values and beliefs. This case highlights that discussions about organ donation should be understood as an integral component of end-of-life care rather than an isolated request. Embedding person-centred nursing practices within intensive care settings may enhance family trust, promote ethical decision-making, and support more humane and dignified experiences at the end of life.
